# OCT2, SSX and SAGE1 reveal the phenotypic heterogeneity of spermatocytic seminoma reflecting distinct subpopulations of spermatogonia

**DOI:** 10.1002/path.2919

**Published:** 2011-08

**Authors:** Jasmine Lim, Anne Goriely, Gareth DH Turner, Katherine A Ewen, Grete Krag Jacobsen, Niels Graem, Andrew OM Wilkie, Ewa Rajpert-De Meyts

**Affiliations:** 1 Weatherall Institute of Molecular Medicine, University of OxfordOxford, UK; 2 Institute of Biological Sciences, Faculty of Science, University of MalayaKuala Lumpur, Malaysia; 3 Department of Cellular Pathology, John Radcliffe HospitalOxford, UK; 4 University Department of Growth and Reproduction, Copenhagen University Hospital (Rigshospitalet)Copenhagen, Denmark; 5 Department of Pathology, Copenhagen University Hospital (Rigshospitalet)Copenhagen, Denmark

**Keywords:** OCT2, SSX2-4, SAGE1, spermatocytic seminoma, spermatogonia

## Abstract

Spermatocytic seminoma (SS) is a rare testicular neoplasm that occurs predominantly in older men. In this study, we aimed to shed light on the histogenesis of SS by investigating the developmental expression of protein markers that identify distinct subpopulations of human spermatogonia in the normal adult testis. We analysed the expression pattern of OCT2, SSX2-4, and SAGE1 in 36 SS cases and four intratubular SS (ISS) as well as a series of normal testis samples throughout development. We describe for the first time two different types of SS characterized by OCT2 or SSX2-4 immunoexpression. These findings are consistent with the mutually exclusive antigenic profile of these markers during different stages of testicular development and in the normal adult testis. OCT2 was expressed predominantly in A_dark_ spermatogonia, SSX2-4 was present in A_pale_ and B spermatogonia and leptotene spermatocytes, whilst SAGE1 was exclusively present in a subset of post-pubertal germ cells, most likely B spermatogonia. The presence of OCT2 and SSX2-4 in distinct subsets of germ cells implies that these markers represent germ cells at different maturation stages. Analysis of SAGE1 and SSX2-4 in ISS showed spatial differences suggesting ongoing maturation of germ cells during progression of SS tumourigenesis. We conclude that the expression pattern of OCT2, SSX2-4, and SAGE1 supports the origin of SS from spermatogonia and provides new evidence for heterogeneity of this tumour, potentially linked either to the cellular origin of SS or to partial differentiation during tumour progression, including a hitherto unknown OCT2-positive variant of the tumour likely derived from A_dark_ spermatogonia. Copyright © 2011 Pathological Society of Great Britain and Ireland. Published by John Wiley & Sons, Ltd.

## Introduction

Spermatogenesis is an ongoing life-long process that begins in fetal development from the specification of primordial germ cells, which are termed gonocytes when they have migrated into the developing testes and become enclosed within seminiferous tubules. During the second and third trimesters of gestation, gonocytes gradually mature to infantile spermatogonia, which acquire competence of transformation into haploid gametes only after puberty. Mature post-pubertal spermatogonia comprise a heterogeneous population of germ cells that can be classified according to their morphologies and correspond to three main maturation stages: A_dark_ spermatogonia, which are considered to represent the reserve stem cell population; highly proliferating A_pale_ spermatogonia; and more mature B spermatogonia that give rise to primary spermatocytes, which enter meiosis and lead to the formation of haploid spermatids [1].

Spermatocytic seminoma (SS) is a rare testicular tumour which was first distinguished from other types of testicular germ cell tumours (TGCTs) that occur in adolescents and young men by Masson (1946) [2]. TGCTs of young men are derived from a precursor condition, called carcinoma *in situ* (CIS) testis or intratubular germ cell neoplasia, unclassified (IGCNU), considered to be derived from developmentally arrested gonocytes [3–6]. By contrast, SS presents in men with a later mean age of diagnosis; ∼54–59 years in most studies [7–9]. However, SS is occasionally diagnosed in men in their thirties or early forties, suggesting that the real age of onset of this benign tumour may be younger than generally believed. SS is a slow-growing neoplasm that exceedingly rarely metastasizes and has a characteristic cytomorphology with the presence of nuclei of three different sizes [9,10]. It is considered to have an early stage of progression in tumourigenesis: the so-called intratubular spermatocytic seminoma (ISS), where abnormal cells accumulate inside the tubules with partially preserved spermatogenesis [11]. This stage should not be confused with CIS/IGCNU, which has a completely different appearance and pathogenesis. Identification of ISS alone—without the presence of a tumour—is extremely rare [9,12] because this lesion is asymptomatic and is not associated with infertility or testicular dysgenesis. Instead, in some SS cases, ISS can be observed adjacent to the invasive tumour. However, it is not clear whether the ISS tubules in the vicinity of the tumour represent the pre-invasive stage of SS or rather a pagetoid spread of invasive tumour within surrounding tubules.

It has been widely agreed that SS differs from other types of TGCTs; however, the identity of the progenitor cells of SS remains controversial. Previous studies have suggested that SS derives from an adult germ cell lineage that lacks any residual embryonic traits, either from primary spermatocytes [13] or from spermatogonia [14]. The pathogenesis has long been unclear, and it was postulated that an amplification of the *DMRT1* locus on chromosome 9 could be involved [13]. Recent molecular analysis of a large panel of SSs provided new insights into the origin of SS, with oncogenic mutations in either *FGFR3* (encoding fibroblast growth factor receptor 3) or *HRAS* (encoding v-Ha-ras Harvey rat sarcoma viral oncogene homolog) present in about 25% of SS specimens [8]. Interestingly, the mutation-positive SSs occurred in a subset of relatively older men (average age 74.9 years, compared with 57.6 years for the mutation-negative samples). These activating *FGFR3* and *HRAS* mutations belong to a category termed paternal-age-effect (PAE) mutations, which are thought to originate from rare random mutational events occurring in the spermatogonia of the normal adult testis. Because of the gain-of-function conferred by the particular mutation within the signal transduction pathway involving FGFR3 and HRAS, mutant spermatogonial cells are proposed to become progressively enriched in the testis over time through a selective proliferation of mutant spermatogonia, leading to clonal expansion which results in the formation of SS tumours in some extreme cases [8].

To gain further insights into the origin and pathogenesis of SS, we studied the protein expression of potential spermatogonial markers during tumour progression and normal testicular development. We selected three candidate markers for detailed study comprising the OCT2 (octamer binding protein 2; also known as POU2F2) transcription factor, which was previously described as being expressed in B cells [15], and two cancer testis antigens, SSX gene family (synovial sarcoma X chromosome breakpoint) and SAGE1 (sarcoma antigen 1) [16,17]. SSX has been previously described in spermatogonia and SS [18,19]; OCT2 and SAGE1 were chosen as potential novel spermatogonial markers after a systematic search of the Human Protein Atlas (http://www.proteinatlas.org) [20]. We report the investigation of these three candidates in a panel of 36 overt SSs, four ISSs, normal adult testis, and a series of testicular tissues representing different stages of germ cell maturation during fetal and childhood development. We found a heterogeneous phenotype of SS and the presence of mutually exclusive subpopulations of spermatogonia in the normal testis, as defined by OCT2 and SSX markers.

## Materials and methods

### Tissue collection

Permission for the anonymous analysis of all human tissue was obtained from the Regional Committee for Medical Research Ethics in Denmark and Oxfordshire Research Ethics Committee A (C03.076) in the UK. The SS samples were collected from the archives of several departments of pathology in Denmark and Sweden. Tumour samples were fixed in 4% buffered formaldehyde and embedded in paraffin. For each SS, representative tumour tissue was identified by a pathologist (GKJ) and used for the preparation of tumour tissue microarrays (TMAs). The TMAs included 36 SS samples, including one with ISS (mean age 63.8 years; range 34–89 years) and control tissues. The latter consisted of five adult testes (morphologically normal tubules in the vicinity of SS), three embryonal carcinomas, one case each of classical seminoma and high mitotic rate seminoma, as well as one sample each of B-cell lymphoma, normal prostate, lung, and epididymis. Each specimen contained a single core of tumour tissue in the TMAs, except two SS samples that were sampled twice. After TMAs were constructed, we also obtained and examined three other ISSs located adjacent to previously identified SSs. Controls additional to those included in the TMAs were three normal adult testes with preserved complete spermatogenesis and without germ cell tumours (obtained following orchidectomy for hernia repair or benign Leydig cell tumour) and a B-cell lymphoma from an adult testis. Twenty-three samples of morphologically normal fetal testicular tissue collected from induced or spontaneous abortion or autopsy of stillbirths ranging from 16th to 42nd weeks of gestation (wg) and without known chromosome or genetic disease, and five samples of infantile and prepubertal testicular tissues (age 2 months–4.5 years) were also included. The tissue examination and determination of the fetal age were performed by an experienced pathologist (NG), as described in detail in previous studies using similar samples [21,22].

### Identification of potential spermatogonial markers from the Human Protein Atlas

OCT2 and SAGE1 were chosen based on two key selection criteria designed to identify potential candidates from the database available in the Human Protein Atlas (http://www.proteinatlas.org). These requirements were strong immunopositivity in cells within the seminiferous tubules of the normal testis and negative expression in other TGCT tissues including embryonal carcinoma and classical seminoma. In addition, the candidates were preferably present in a subpopulation of spermatogonia in the normal adult testis.

### Immunohistochemistry

#### Antibodies

The antibodies used were mouse monoclonal (MAb) anti-OCT2 (clone Oct-207; Novocastra Laboratories Ltd, Newcastle, UK); mouse MAb anti-SSX, which recognizes SSX2, SSX3, and SSX4 [18], thus it is thereafter named SSX2-4 (clone E3AS; a kind gift from Professor Dr A Geurts van Kessel, Department of Human Genetics, University Hospital, Nijmegen, The Netherlands); rabbit polyclonal (PAb) anti-SAGE1 (code HPA003033; Sigma-Aldrich, Pool, UK); mouse MAb anti-Ki67 (clone MIB-1; Dako, Glostrup, Denmark); rabbit PAb anti-GPR125 (G protein-coupled receptor 125) (codes ab51705 and GB-10 400; Abcam, Cambridge, UK and Genesis Biotech Inc, Taipei, Taiwan, respectively); mouse MAb anti-OCT3/4 (clone C-10; Santa Cruz Biotechnology Inc, Santa Cruz, CA, USA); mouse MAb anti-PLAP (placental-like alkaline phosphatase) (clone 8A9; Dako, Glostrup, Denmark) and mouse MAb anti-MAGEA4 (clone 57B; a kind gift from Professor Giulio C Spagnoli, Department of Urology, University Hospital of Zurich, Zurich, Switzerland). Staining with anti-MAGEA4 was used as a control for the integrity of tissues, due to its strong expression in SS and spermatogonia. All antibodies have been extensively validated and optimized for immunohistochemistry in previous studies [18,20,22,23,24]. The test results with GPR125, previously described as a surface marker of spermatogonia [23], showed non-specific high cytoplasmic background staining after incubating at different dilutions with both antibodies, so studies with this marker were not pursued further.

#### Single immunostaining procedure

Five-micrometre deparaffinized and rehydrated sections were processed for immunohistochemistry. All incubations were performed at room temperature unless stated otherwise. For antigen retrieval, microwave heat treatment was conducted in citrate buffer (10 mm, pH 6) for 16 min. Samples were allowed to cool to room temperature before incubation with 3% H_2_O_2_ in Tris-buffered saline (TBS) for 10 min to quench endogenous peroxidase activity. Subsequently, sections were incubated overnight at 4 °C with primary antibodies including anti-OCT2 (1:100), anti-SSX (1:100), anti-SAGE1 (1:3000), anti-GPR125 (1:100–1:5000), anti-Ki67 (undiluted) anti-PLAP (1:100), anti-OCT3/4 (1:100), and anti-MAGEA4 (1:500). For negative controls, primary antibodies were replaced with TBS. Primary antibodies were detected using the EnVision + DualLink System, Peroxidase (DAB+) kit (code K5007, Dako), according to the manufacturer's recommendations. Staining was completed by a 10 min incubation with 3,3′-diaminobenzidine (DAB+) substrate–chromogen buffer before counterstaining with haematoxylin. The staining was assessed independently by three investigators (JL, GDHT, and ERDM) using a systematic semi-quantitative scoring system based on the approximate proportion of cells stained and the staining intensity (adapted from a previous study [25]), with the exception of Ki67 (a cell proliferation marker), where the numbers of stained cells were counted in one to four representative areas (in most cases two, because the core size was quite small) comprising approximately 100 cells per area. An unbiased estimate of the standard deviation (SD) was calculated using Microsoft Excel when more than two counts were taken. The proportions of large, intermediate, and small cells were similarly quantitated. Statistical comparisons of staining patterns, age of the subjects with SS, and mutation status of the SS employed two-tailed Fisher's exact test or Student's *t*-test as appropriate.

#### Double immunostaining procedure

Sequential double-staining for OCT2 and SSX2-4 (or OCT3/4, PLAP) was performed using the EnVision™ G| 2 Doublestain System, Rabbit/Mouse (DAB + /Permanent Red) kit (code K5361, Dako), according to the manufacturer's recommendations. Following heat-induced epitope retrieval, the endogenous peroxidase, alkaline phosphatase (AP), and pseudo-peroxidase activities were inhibited with Dual Endogenous Enzyme Block reagent and sections were incubated with the OCT2 antibody overnight at 4 °C. After the detection with Polymer/HRP (horseradish peroxidase) and visualization by DAB+ chromogen, the sections were blocked with Doublestain Block reagent before incubation with SSX, PLAP or OCT3/4 antibody at 4 °C overnight. Sections were incubated with Rabbit/Mouse (LINK), followed by Polymer/AP, and the reaction was visualized with Permanent Red chromogen. All the specified reagents, except the primary antibodies, were provided with the kit. Double-stained sections were then counterstained with haematoxylin and mounted with Aquatex® (Merck KGaA, Darmstadt, Germany). Washing steps using TBS/Tween-20 buffer were conducted between each step of antibody incubation, detection, and colour development.

### Mutation analysis of *FGFR3* and *HRAS* in tumour associated with ISS

DNA was extracted from 5 µm paraffin-embedded sections of bulk tumour present in three ISS samples (ISS2, ISS3, and ISS4) using the Nucleon® Genomic DNA Extraction Kit for hard tissue (code SL 8509; Tepnel Life Sciences, Manchester, UK) according to the manufacturer's recommendations. Tumour samples were screened for mutations in exons 15 and 3 of the *FGFR3* and *HRAS* genes respectively, using the previously described PCR primer pairs and conditions [8]. Mutations previously identified in SS [8] were sought in the amplified products by restriction digestion with *Alw26I* (1948G+) or *BpI1* (1948G−) (Fermentas, St. Leon-Rot, Germany) for the *FGFR3* 1948A > G substitution and *BstNI* (New England Biolabs, Ipswich, Massachussets, USA) for the *HRAS* 181C > A and 182A > G substitutions. Digestion products were analysed on 3% agarose gels.

## Results

### Analysis of the ontogeny of OCT2, SSX2-4, and SAGE1 expression in normal testis

**Figure 1:**
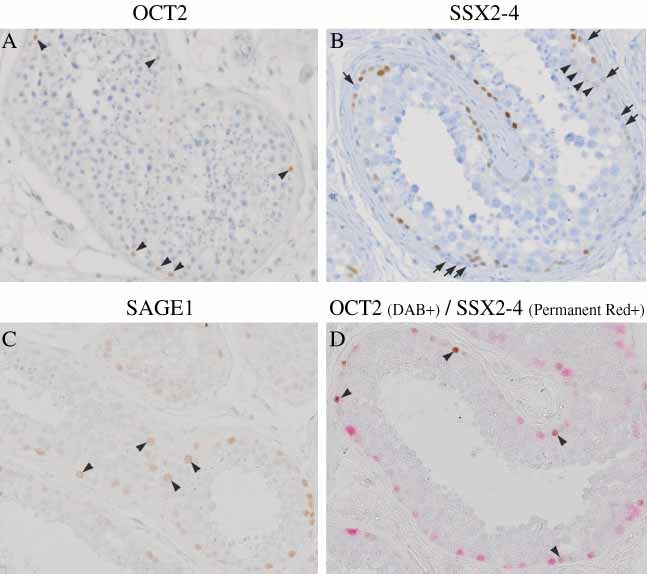
Distribution of OCT2, SSX2-4, and SAGE1 in the normal adult testis. (A) OCT2: note the strong nuclear staining in a minority of spermatogonia located adjacent to the basement membrane of the tubules (arrowheads). (B) SSX2-4 was detected mainly in A_pale_/B spermatogonia but was absent in A_dark_ (arrows) spermatogonia. Some of the primary spermatocytes (arrowheads) showed weak staining. (C) SAGE1 was found predominantly in B spermatogonia, characterized by a large circular shape and more internal position in tubule (arrowheads). (D) OCT2 and SSX2-4 double-staining revealed that OCT2 (brown nuclei) and SSX2-4 (red nuclei) were present in two distinct subpopulations of spermatogonia. OCT2 was mainly found in the A_dark_ spermatogonia (arrowheads). Scale bar = 100 µm.

In the normal adult testis, OCT2, SSX2-4, and SAGE1 nuclear staining was detected in distinct subpopulations of spermatogonia (Figures 1A–1C). GPR125 did not yield satisfactory staining (see Materials and methods section) and was not analysed in detail.

OCT2 was present predominantly in the A_dark_ spermatogonia recognized by the presence of the vacuole-like cavity in the nucleus, whilst the immunoreactivity of SSX was detected mainly in A_pale_/B spermatogonia, with a weak reaction also observed in a subset of leptotene spermatocytes. SAGE1 was seen in a small subset of A spermatogonia but was localized predominantly to more differentiated B spermatogonia, distinguished by their larger and more circular nucleus compared with A_dark_ and A_pale_ spermatogonia (which have smaller, ovoid nuclei). Notably, the staining pattern of OCT2 and SSX2-4 in spermatogonia was mutually exclusive and this was further confirmed by dual immunohistochemistry (Figure 1D).

**Figure 2:**
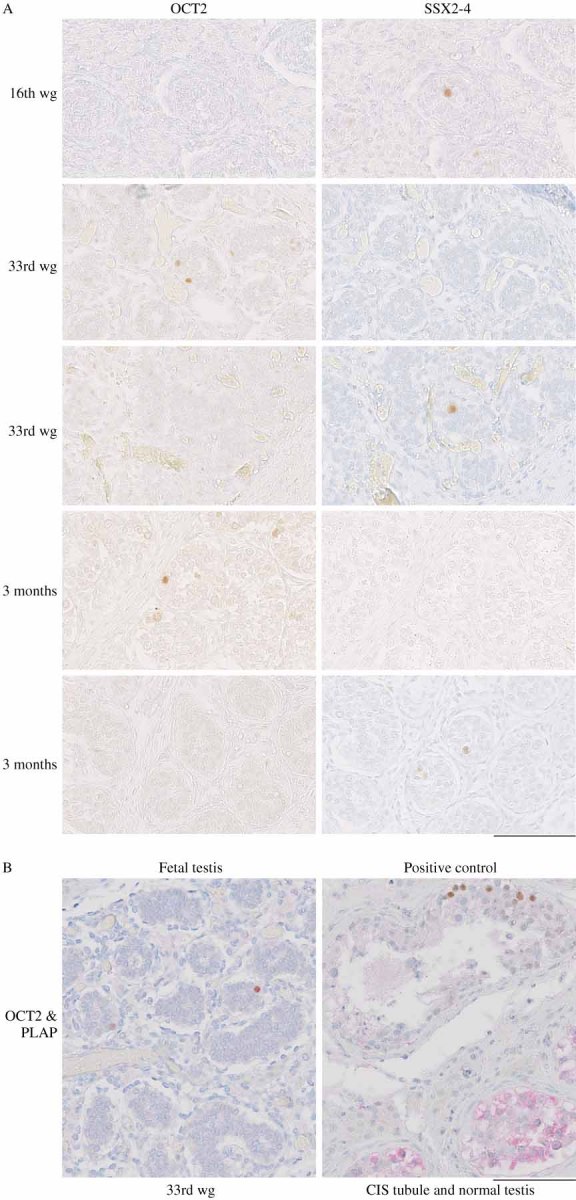
Comparative staining of OCT2, SSX2-4, and PLAP in distinct germ cell subpopulations during testis development. (A) The presence of SSX2-4 was first noted at the 16th wg. OCT2 and SSX2-4 are expressed distinctively in two different subsets of germ cells, starting from the 33rd wg to adulthood including at the age of 3 months. Note that horizontally paired images, taken from adjacent sections of the same testis, allow comparison of the staining of individual germ cells for each protein. (B) Left: double labelling of fetal testis at the 33rd wg demonstrates that PLAP is not detected in germ cells expressing OCT2 (brown nuclei). Right: positive control showing strong membranous and cytoplasmic immunoreactivity of PLAP in carcinoma *in situ* (CIS) cells (red) and OCT2-positive spermatogonia (brown) in the adjacent tubule. Scale bars = 100 µm (all images).

We extended our study to outline the antigenic profile of OCT2, SSX, and SAGE1 during different stages of testis development. In our series starting from the 16th week of gestation (wg) to 4.5 years, we found that OCT2 was present in most of the cases studied. However, OCT2 was not detected in gonocytes, which were identified by double staining with OCT3/4 or PLAP protein (Figure 2B). As a control for tissue integrity, MAGEA4 was detected in the prespermatogonia from the 20th wg onwards, but not in the gonocytes, which express OCT3/4 and PLAP, in agreement with the previously established pattern [21,22,26]. In mid-gestation, we identified single germ cells (possibly prespermatogonia) with strong OCT2 expression only in the 17th and 18th wg. By the 33rd wg, the intense OCT2-immunopositive cells reappeared and these cells were then consistently observed in postnatal testicular tissue. Only a few representative cases were investigated for SSX because this antigen has been studied previously [19]. Weak expression of SSX2-4 was first noted at the 16th wg but a subset of cells showing strong immunoreactivity was found from the 21st to the 33rd wg and also in testicular tissue from a 3-month-old child. By comparing adjacent serial sections (Figure 2A), we established that SSX2-4 and OCT2 immunopositive staining was always observed in mutually exclusive subsets of germ cells. The expression of SAGE1 protein was absent at all ages during pre-pubertal development, suggesting that up-regulation of this protein occurs after puberty.

### Analysis of OCT2, SSX2-4, and SAGE1 expression in spermatocytic seminoma and intratubular spermatocytic seminoma

**Figure 3:**
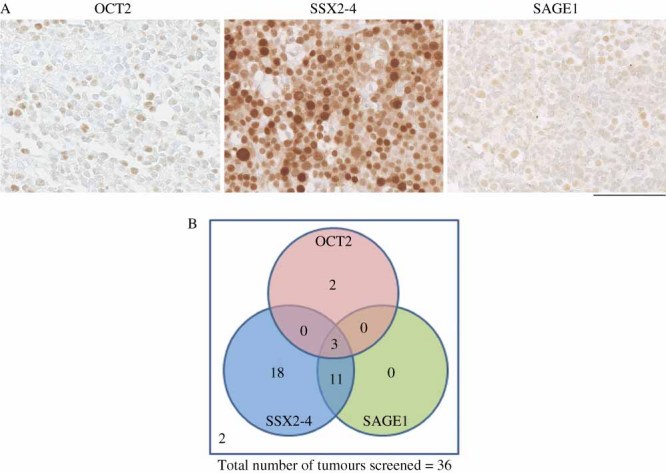
Expression of OCT2, SSX2-4, and SAGE1 in spermatocytic seminoma (SS). (A) Examples of immunohistochemical detection of OCT2 (left), SSX2-4 (middle), and SAGE1 (right) in SS. A heterogeneous nuclear staining pattern with different intensities is seen in the tumour cells. Scale bar = 100 µm. (B) Venn diagram showing the relationship between OCT2, SSX2-4, and SAGE1 expression in SS.

Having established the pattern of expression in the normal testes, we analysed the expression of the candidate proteins OCT2, SSX2-4, and SAGE1 in 36 SS cases as summarized in Table 1. The results (examples shown in Figure 3A) revealed that these proteins were all expressed, with variable intensities, in subpopulations of tumour cells in a proportion of SSs. Most tumours (32; 89%) were positive for SSX2-4 and amongst these, 14 (39%) were positive for SAGE1. OCT2 was expressed in a much smaller proportion of tumours (5; 14%), all of which were either positive for SSX2-4 and SAGE1 or negative for both markers (Figure 3B); there was a negative trend between OCT2 and SSX2-4 expression, although this did not achieve formal statistical significance (*p* = 0.08, Fisher's exact test). The two OCT2^+^/SSX2-4^−^/SAGE1^−^ samples were negative for FGFR3 and exhibited a relative paucity of large cells (which in OCT2-negative samples ranged from 0.6% to 2.7% of the total cell population). The proliferation index based on Ki67 staining was very variable in all SS samples and did not correlate with the expression of any of the three markers (Table 1).

**1:** Summary of the immunohistochemical analysis in relation to the mutation status in the series of spermatocytic seminoma (SS)

Case No*	Age (years)	Mutation found*	OCT2	SSX2-4	SAGE1	Ki67 (% positive ± SD)
SS4	75	−	Neg	+ (w)	Neg	7.0, 11.5
SS5	76	−	Neg	+ (w)	Neg	42.2†
SS7	50	−	Neg	+ + (str)	Neg	n/a
SS8	86	−	Neg	+ + + (str)	+ (w)	0, 9.1
SS9	87	*FGFR3* (K650E)	Neg	+ (w)	Neg	5.4, 0.9
SS10	61	−	Neg	+ + (str)	Neg	6.4, 6.8
SS11	64	−	+ (w)	Neg	Neg	24.2†
SS13	34	−	+ (w)	+ + (str)	+ + + (str)	4.3, 5.4
SS14	71	n/r	Neg	+ (str)	+ (w)	0, 0.9
SS15	53	−	+ (w)	+ + + (str)	+ + (str)	8.1, 6.1
SS16	40	−	Neg	+ + + (str)	Neg	30.8, 26.1
SS17	61	n/r	Neg	Neg§	Neg	18.5, 19.8
SS19	44	−	Neg	+ + (str)	+ (w)	3.3, 17.2
SS20	37	−	Neg	+ + (str)	Neg§ (w)	15.8, 19.6
SS21	67	*HRAS* (Q61R)	Neg	+ + + (str)	+ (w)	28.6, 13.7
SS22	41	−	Neg	+ (w)	Neg	46.0, 59.5
SS23	69	*HRAS* (Q61R)	Neg	+ + (str)	Neg	44.3, 43.2
SS24	79	n/r	Neg	+ (w)	+ + (w)	13.5, 7.6
SS25	89	n/r	Neg	+ + (str)	Neg	0, 0‡
SS26	n/a	−	Neg	+ + + (str)	+ + (str)	31.5, 24.4
SS27	55	−	Neg	+ + + (str)	+ + + (str)	22.8, 17.2
SS28	63	n/r	Neg	+ (str)	+ + + (str)	n/a
SS29	75	n/r	Neg	+ (str)	Neg	2.2, 0
SS30	79	*HRAS* (Q61K)	Neg	+ + + (str)	Neg	0, 1.1
SS31	75	*FGFR3* (K650E)	Neg	+ + + (str)	Neg	32.1, 44.2
SS32	44	n/r	+ (w)	+ + (str)	+ + + (str)	9.2 ± 3.5
SS33	52	−	Neg	+ (w)	Neg	2.7, 6.0
SS35	80	*HRAS* (Q61R)	Neg	+ + + (str)	Neg	22.0, 19.0
SS37	47	−	Neg	+ + + (str)	+ + (str)	46.4, 39.1
SS38	74	−	+ + (str)	Neg	Neg	51.3, 62.0
SS39	43	n/r	Neg	+ + + (str)	+ + (w)	24.8, 23.5
SS40	67	*HRAS* (Q61K)	Neg	+ + (str)	Neg	6.2, 2.9
SS41	61	n/r	Neg	Neg	Neg	3.8, 2.9
SS42	73	n/r	Neg	+ + (w)	Neg	15.5, 9.6
SS43	87	n/a	Neg	+ + (str)	+ (w)	n/a
SS44	75	n/a	Neg	+ (w)	Neg	n/a

* Most of the tumours were investigated for candidate gene mutations in the previous study [8], so the original numbering is retained.

† Only a very small number of cells were available in the tissue core.

‡ Ki67 antigen negative but some mitotic figures visible in the core.

−, no mutation found; n/r, no results owing to the low quality of tumour DNA; n/a, not available. Staining score describes the proportion of positive cells as + + +, nearly all cells stained; + +, approximately half of the cells stained; +, a low percentage of cells stained; Neg, no positive cell detected; or

§ only single cells stained. Staining intensity is categorized as str, strong; or w, weak. Ki67 results are presented as a percentage (%) of stained cells, when one to two separate counts were taken, or as % of stained cells ± unbiased estimate of SD, when four separate counts were taken (staining intensity was strong in all specimens).

**Figure 4:**
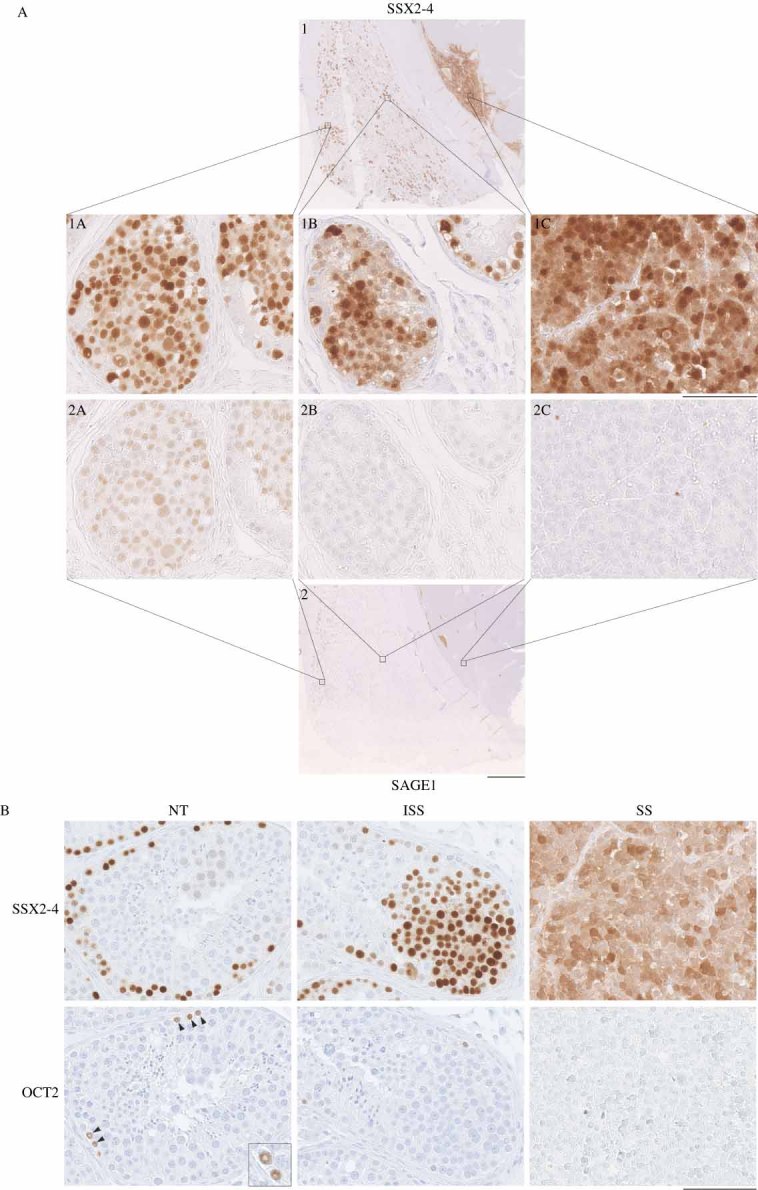
The distribution of spermatogonial markers during progression in two cases of intratubular spermatocytic seminoma (ISS). (A) ISS4. Sections labelled 1 and 2 provide a low-power (scale bar = 2.5 mm) overview of adjacent sections stained with SSX2-4 and SAGE1, respectively. A subpopulation of ISS tumour cells co-expressing both SSX2-4 and SAGE1 (1_A_, 2_A_) is mainly found in a region located away from the bulk of the tumour (starting from the edge of the sections). However, SAGE1 is absent and only SSX2-4 is observed in the ISS cells in close vicinity of the tumour (1_B_, 2_B_). In the overt SS region, focal SSX2-4 expression is found (1_C_) and no SAGE1-immunopositive cells are detected (2_C_). Scale bar = 100 µm. (B) ISS2 showing the presence of normal tubules (NT), ISS, and SS within a single section. Note the positive staining for SSX2-4, but negative staining for OCT2, within both SS and ISS. However, occasional cells (A_dark_ spermatogonia shown in the inset) are OCT2-positive in adjacent normal tubules (arrowheads). Scale bar = 100 µm.

To gain further insight into the cell populations expressing OCT2, SSX2-4, and SAGE1, we examined their expression profiles in ISS. Our analysis is based on only four cases (Table 2), owing to the apparent rarity with which this histological pattern is recognized. This revealed that most of the ISS cells clustering within the seminiferous tubules were either positive for SSX2-4 only or co-expressed SSX and SAGE1 proteins (Table 2). A heterogeneous staining pattern suggesting tumour evolution was identified within two of the four ISS cases studied (Table 2 and Figure 4A). There were some regions where ISS cells only expressed SSX2-4 but not SAGE1, while co-expression of both proteins was found in other regions. In the region closer to the overt SS, the expression of SAGE1 appeared to be gradually reduced and was totally undetectable in the cells located in close vicinity of the bulk of the tumour. However, SSX2-4 remained present even in cells close to the tumour and was found focally in the overt SS. OCT2 was found occasionally in the spermatogonia within the normal seminiferous tubule but was absent from the precursor stage of SS and the overt tumour in these four cases (Figure 4B and Table 2).

**2:** Intratubular spermatocytic seminoma (ISS) samples included in the study. Staining score and the Ki67-based proliferation score are the same as in Table 1

Case No	Age (years)	Mutation found	Cell type	OCT2	SSX	SAGE1	Ki67 (% positive ± SD)
ISS1	75	*FGFR3* K650E	Overt SS (SS31)	−	+ + +	−	32.1, 44.2
			ISS	−	+ + +	+ +	36.6, 25.8
			SPG (adjacent tubule)	+	+ + +	+	n/d
ISS2	68	−	Overt SS	−	+ + +	+ + +	39.6 ± 7.4
			ISS	−	+ + +	+ + +	19.1 ± 2.9
			SPG (adjacent tubule)	+	+	+	n/d
ISS3	31	−	Overt SS	−	+ + +	+ + +	22.9 ± 4.4
			ISS	−	+ + +	+ + + (focal)	15.3, 18.5
			SPG (adjacent tubule)	+	+	+	n/d
ISS4	36	−	Overt SS	−	+ + + (focal)	−	13.1 ± 7.1
			ISS	−	+ +	+ + + (focal)	15.3, 5.0
			SPG (adjacent tubule)	+	+	+	n/d

SPG = spermatogonia. n/d = not done

### Mutational analysis of samples with intratubular spermatocytic seminoma (ISS)

The mutation status in 24 SS cases (Table 1) was previously investigated for 15 different candidate genes [8]. All the tumours identified with *FGFR3* or *HRAS* mutations expressed SSX2-4 (7/7), whereas all OCT2-positive tumours were mutation-negative (4/4); however, these differences were not statistically significant. One sample that included a region of ISS (ISS1) was positive for the *FGFR3* mutation 1948A > G encoding K650E. We screened the bulk of tumour present in three additional ISS cases for activating mutations in *FGFR3* and *HRAS*, but no sequence variations were identified in these samples (Table 2).

## Discussion

Based on the analysis of three spermatogonial markers OCT2, SSX2-4, and SAGE1, we report for the first time the phenotypic heterogeneity of SS. Moreover, we identified the expression of OCT2 and SSX2-4 in distinct subpopulations of spermatogonia in the normal adult testis as well as during testicular development, thus adding to the understanding of the phenotypic changes of the germ cell during maturation.

OCT2 is a transcription factor from the POU (Pit-1, Oct1/2, UNC-86) family that shares a conserved 150–160 amino acid domain [27]. Expression of OCT2 has been described in B lymphocytes, neuronal cells, activated T cells, and macrophages [15,28–30]. This antigen was also highly expressed in two B-cell lymphomas used in this study. It is interesting to note that one of these samples was previously misdiagnosed as SS and was reclassified by one of the authors (GKJ), partly based on the antigen profile. The presence of OCT2 in the normal adult testis was initially identified by a systematic analysis of proteins' expression performed on a series of normal tissues in the Human Protein Atlas (http://www.proteinatlas.org) [20]. Another member of this family, OCT3/4, has been studied extensively in the human testis and its expression has been documented during embryonic germ cell maturation [22,26,31] and in various testicular tumours [4]. OCT6, a third family member, has been implicated as an intrinsic regulator for GDNF (glial cell line-derived neurotrophic factor)-induced survival and self-renewal in mouse spermatogonial stem cells [32] but has not been studied in human testis. OCT2, on the other hand, has not been investigated in the testis of any other species. Notably, OCT2 was absent in gonocytes positive for OCT3/4 or PLAP, indicating that it is specific for more mature germ cells. The identification of OCT2 as a potential marker of A_dark_ spermatogonia, the reserve population of germ cells, is one of the most interesting findings in this study. It may have some clinical value for a more objective assessment of the recovery of spermatogenesis and fertility potential, for example in cancer survivors or young individuals with a history of cryptorchidism.

Since different subsets of human spermatogonia were first described [33], many studies have been performed to identify markers that are potentially specific for these subtypes of spermatogonia; as reviewed in ref 34. In this study, we were able to distinguish the presence of different subpopulations of spermatogonia in the normal adult testis using the combination of OCT2, SSX2-4, and SAGE1 markers. Following this observation, further investigations were performed to understand their expression patterns during early testicular development. Strong expression of OCT2 and SSX2-4 in germ cells was seen at different stages of germ cell maturation. Intriguingly, OCT2 and SSX2-4 were present in two mutually exclusive subsets of germ cells, suggesting that these proteins play distinctive roles at different stages during normal testicular development. Most of the cells expressing OCT2 and SSX2-4 were located adjacent to the basement membrane, although some cells with strong SSX2-4 immunopositivity were observed in the centre of the seminiferous cord.

SSX proteins and SAGE1 (as well as our control antigen, MAGEA4) are cancer testis antigens encoded by genes that are normally only expressed in human germ cells, trophoblast, and certain tumours [35]. It has been suggested that SSX genes act as repressors in transcriptional regulation [36]. Transcripts encoding several cancer testis antigens including the SSX family and SAGE1 were ranked among the top 50 discriminators specifically expressed in SS [13]. However, the function of these proteins in either spermatogenesis or tumourigenesis remains elusive. SAGE1 was absent in the fetal and infantile testes, and based on its expression in normal adult testis, we hypothesize that this protein is up-regulated in spermatogonia only after puberty.

In the present study, the expression profile of OCT2, SSX2-4, and SAGE1 proteins provides further evidence to support the spermatogonial origin of SS. Although a previous study suggested primary spermatocytes as the origin of this tumour [13], our analysis confirmed that SS is most likely derived from different subtypes of spermatogonia, which can be identified by OCT2 and SSX2-4 markers. This finding is consistent with the antigenic profile of these markers in the normal adult testis (as mentioned above). Notably, the hitherto unknown OCT2-positive SS represents a relatively rare subtype of this tumour (14%; 5/36); therefore, previous comprehensive profiling of SS may have failed to identify this marker [13]. Alternatively, the expression of OCT2 may be a sign that some spermatogonia reverted to a less mature A_dark_, but this is less likely, because Adark spermatogonia divide rarely and are considered a reserve population. In addition, SAGE1 was identified in a subset of SSX2-4-expressing SSs, indicating an origin from the same cell type but potentially at a different stage of tumour progression. This was further investigated in SS samples that included ISS, where at least in some tubules the morphology of the cells suggested a transition between normal germ cells and SS tumour cells, indicating that these ISS forms were indeed an early, pre-invasive stage in SS progression. In two of these rare cases, we identified spatial differences of SSX2-4 and SAGE1 expression in different subpopulations of ISS cells, with the invasive tumour expressing only SSX antigen (Figure 4A). The gradual loss of SAGE1- and gain of SSX2-4-positive cells (the first expressed in spermatogonia; the latter in spermatogonia and early spermatocytes) in one ISS specimen with morphological signs of transformation may suggest that a subset of the transformed cells mature further towards pre-leptotene spermatocytes, which still express at least one of the SSX proteins. This is consistent with the known plasticity of neoplastic germ cells, which may be at different stages of differentiation, and would explain the expression in SS of some genes that are also expressed in early spermatocytes [13]. Of note, SSX antibody recognizes three different SSX antigens [18] and there may be subtle differences in their expression patterns within germ cell maturation stages; all genes were represented in overt SS at the mRNA level [13] but it is not known whether all are translated.

Taken together, the distinct temporal pattern of expression of OCT2, SSX2-4, and SAGE1 proteins, with mutual exclusivity of OCT2 and SSX2-4, demonstrates the existence of different populations of spermatogonia not only in the adult testis, but also before puberty. These findings also explain the observations that SS may have its origins in different spermatogonial cell populations characterized by either SSX2-4 or (in a minority) OCT2 expression. We suggest that SS is derived predominantly from the more mature spermatogonia (A_pale_ or B spermatogonia), as proposed in previous studies [14,37], and a relatively small subset of this tumour type may derive from A_dark_ spermatogonia. However, the observed phenotypic heterogeneity of SS may reflect changes of gene expression occurring during tumour progression. Most of the subtypes of SS, except those with an OCT2^+^/SSX2-4^−^/SAGE1^−^ expression pattern, which seem to be locked in this phenotype, seem to retain plasticity and the ability to mature partially towards early spermatocytes, at least in a subset of tumour cells.

In conclusion, our data provide a new understanding of the histogenesis and progression of SS, which appears to be a more heterogeneous tumour type than previously thought. This is also the first documented evidence of the expression profile of OCT2 and SAGE1 in human testis from embryonic development to adulthood. In addition, this study has highlighted the presence of two mutually exclusive subpopulations of germ cells defined by OCT2 or SSX protein expression. Future studies will be necessary to confirm whether OCT2 is indeed a specific marker for human reserve spermatogonial stem cells, which could have potential clinical use, and to uncover its role in germ cell development and spermatogenesis.
